# The alteration of intrauterine microbiota in chronic endometritis patients based on 16S rRNA sequencing analysis

**DOI:** 10.1186/s12941-023-00556-4

**Published:** 2023-01-12

**Authors:** Qing Chen, Xiaowei Zhang, Qicai Hu, Wei Zhang, Yi Xie, Weixia Wei

**Affiliations:** 1 Department of Obstetrics and Gynecology, Peking University Shenzhen Hospital, Shenzhen, 518036 P. R. China; 2 Institute of Obstetrics and Gynecology, Shenzhen PKU-HKUST Medical Center, Shenzhen, 518036 P. R. China; 3 Shenzhen Key Laboratory on Technology for Early Diagnosis of Major Gynecologic Diseases, Shenzhen, 518036 P. R. China

**Keywords:** Chronic endometritis, Intrauterine microbiota, 16S rRNA sequencing, Firmicutes (Bacillota), *Lactobacillus*

## Abstract

**Background:**

Chronic endometritis (CE) is a disease of continuous and subtle inflammation occurring in the endometrial stromal area, which is often asymptomatic or present with non-specific clinical symptoms.

**Methods:**

This study investigated the composition and distribution of the intrauterine microbiota of 71 patients who underwent hysteroscopy during the routine clinical inspection of infertility. Among them, patients who were diagnosed with chronic endometritis (CE) were allocated into CE group (n = 29) and others into non-CE group (n = 42). There was no significant difference in average age between the two groups (P = 0.19). Uterine flushing fluid was collected by the self-developed cervical trocar uterine cavity sampler and 16S rRNA sequencing was performed.

**Results:**

The alpha diversity in the CE group was significantly higher than that in the non-CE group (P < 0.05). Firmicutes (newly named Bacillota) were the dominant phylum in the non-CE group (72.23%), while their abundance was much lower in the CE group (49.92%), but there was no statistically significant difference between the two groups. The abundances of Actinobacteriota and Cyanobacteria in the CE group were significantly higher than those in the non-CE group (P < 0.05). At the genus level, the abundance of *Lactobacillus* dominated in all samples, which presented a significantly lower abundance in the CE group (40.88%) than that in the non-CE group (64.22%) (P < 0.05). Correspondingly, the abundance of non-*Lactobacillus* was higher in the CE group, among which *Pseudomonas* and *Cutibacterium* increased significantly (P < 0.01). Moreover, compared with the non-CE group, the pathways involved in arginine and proline metabolism and retinol metabolism were significantly enriched in the CE group (P < 0.05), while the metabolism of lipid and prenyltransferases were significantly decreased in the CE group (P < 0.05).

**Conclusions:**

A certain microbial community was colonized in the uterine cavity, which was dominated by *Lactobacillus*. The structure and distribution of intrauterine microbiota in the CE group were different from those in the non-CE group by showing a lower abundance of *Lactobacillus*, and a significantly higher abundance of *Pseudomonas* and *Cutibacterium*. Additionally, the microbial metabolism was altered in the CE group. This study elaborated the alteration of intrauterine microbiota in CE patients, which may contribute to the diagnosis of CE and provide a reference for antibiotic treatment of CE.

## Background

Chronic endometritis (CE) is a disease of continuous and subtle inflammation occurred in the endometrial stromal area, which is often asymptomatic or present with non-specific clinical symptoms, such as pelvic pain, dysfunctional uterine bleeding, dyspareunia, vaginitis, etc. Studies have shown that CE is associated with infertility, repeated abortion and repeated implant failure [1–3]. As a result, most of the CE cases were diagnosed by the routine examination of infertility patients, and the prevalence rate is as high as 67%. Currently, microbial infection is regarded as one of the main causes of CE [1, 2]. Previous studies have confirmed that the administration of antibiotics is an effective treatment method for CE which can improve fertility outcomes significantly [4, 5].

With the rapid development of sequencing technology, more accurate molecular technology has been used for the diagnosis of CE toward pathogenic microorganism detection. On the other hand, with the improvement of intrauterine sampling technology, the traditional concept of sterile uterine cavity has been broken [6]. It has been elaborated that a complex bacterial community was colonized in the uterine cavity. Although the biomass of the community is relatively low, they keep a balanced intrauterine microenvironment through bacterial metabolisms and interaction. The pathogenic invasion may disrupt the balance, shape the microbial community, and may further cause endometritis. However, rare studies were focused on the specific characteristics of the disordered microbiota in the uterine cavity of CE patients. An explanation of microbial community based on a large sample size will contribute to the precise diagnosis and treatment of CE.

In this study, 16S rRNA gene sequencing technology was used to study the microbiota in the uterine cavity of infertile patients with CE. The community composition and distribution were characterized to provide a basis for improving the diagnosis rate of CE and guiding the selection of antibiotic treatment for CE patients.

## Methods

### Participates

Patients who underwent hysteroscopic surgery for the routine infertility examination at Peking University Shenzhen Hospital (Shenzhen, China) between January 2019 and December 2020 were recruited in this study. Among them, the patients who did not receive vaginal medication and had no sexual behaviors within 7 days after menstruation, and met the following inclusion and exclusion criteria were informed and enrolled. The inclusion criteria were as follows: (1) patients who under went in vitro fertilization and embryo transfer (IVF-ET) and failed no less than one time; (2) patients who experienced spontaneous abortion no less than two times; (3) patients who were diagnosed with infertility. The exclusion criteria were as follows: (1) patients with acute and chronic infectious diseases, diabetes, cardiovascular diseases, autoimmune diseases, thyroid diseases and mental diseases; (2) patients who have not received contraceptive or steroid treatment within six months and have not received systemic antibiotics within three months. This study was approved by the Ethics Committee of Peking University Shenzhen Hospital (No. 2019–064), and all participants provided written informed consent.

### Sampling and hysteroscopic examination

During the surgery, the vulvovaginal was sterilized routinely after intravenous general anesthesia, and then a disposable sterile vaginal speculum was inserted into the vagina. The self-developed transcervical cannula uterine cavity sampler (Patent Number: ZL 2018 2 1945014.0) was used to avoid the contamination of vaginal and cervical secretions. Firstly, a disposable sterile external cannula was placed in the cervical canal. Secondly, we connected a uterine cavity sampling catheter to a 10 ml syringe, which was pre-injected with 3 ml sterile saline, and inserted into the cannula. After injecting the saline for 1 min, the solution was extracted by turning the uterine cavity clockwise for a circle. Then, the sampling catheter was returned back to the cannula and removed together. Finally, the sample was injected into a sterile EP tube, and stored in a – 80 °C refrigerator. Also, the uterine manifestations and endometrial biopsy were described according to the criteria of hysteroscopy for CE diagnosis [7].

### Histological analysis and immunohistochemistry

Endometrial samples were fixed in neutral formalin and embedded in paraffin for histological analysis and immunohistochemistry. All biopsy blocks were serially sectioned at a thickness of 3–4 μm and incubated with mouse anti-human monoclonal CD138 antibody (Biocare Medical, Concord, CA, USA) and the secondary antibody used was a labeled polymer horseradish peroxidase anti-mouse antibody (Maixin, Fuzhou, China) following the protocol. The number of CD138+ cells more than 4/HPF + was diagnosed as CE [7–9].

### DNA extraction and 16S rRNA amplicon sequencing

We sequenced the samples from cervix samples by amplicon sequencing of partial 16S rRNA gene [10]. The genomic DNA from samples was extracted by QIAamp^®^ DNA Mini Kit (Qiagen, Hilden, Germany). The extracted product was used as a template to amplify the 16S rRNA V4 region after passing the quality control of nanodrop (Thermo Fisher Scientific, Waltham, MA, USA), Qubit 3.0 (Invitrogen, Carlsbad, CA, USA), and agarose gel electrophoresis. The V4 region of the 16S rRNA genes was amplified by PCR with universal primers V4-515F (5ʹ-GTGCCAGCMGCCGCGGTAA-3ʹ) and 806R (5ʹ- GGACTACHVGGGTWTCTAAT -3ʹ). After the PCR product was detected and purified by agarose gel electrophoresis and the concentration was qualified, the library has obtained after polyadenylation. The Qubit 3.0 Fluorometer and Agilent 2100 Analyzer were used for quality control of the constructed library. Subsequently, the total DNA was sequenced by the Illumina Mini Seq platform for pair-end 300 bp sequencing.

### Bioinformatic analysis

The newly generated raw sequencing data were processed for quality control using the FASTX toolkit. QIIME2 software was used to remove sequenced primers and barcodes, obtain chimeric artifacts, calculate Bray–Curtis distance matrix construction, and pick Operational Taxonomic Units (OTUs) [11, 12]. The sequences of the remaining libraries were grouped into OTUs based on 97% similarity using the UCLUST program. The seeded sequences of each OTU were chosen for taxonomic classification against the Silva 138 SSURef NR99 full-length sequences [13] by the UCLUST taxonomy assigner. The relative abundance profiles of the main phylum and genus were quantified. STAMP software was invited to predict the functional profiles of the microbial communities [14].

### Statistical analysis

SPSS 22.0 software was used for statistical analysis. The continuous variable data were confirmed as a normal distribution before *T*-test was applied. The categorical variable data were presented as percentages and the χ2 test was used. P < 0.05 was considered statistically significant.

## Results

### No significant difference was found in the clinical characteristics between CE and non-CE groups

A total of 71 participants were included, with an average age of 31.77 ± 3.88 years. Among them, 29 cases with chronic endometritis (CE group) and 42 cases of non-chronic endometritis (non-CE group) were identified according to the results of histophiological and immunohistochemical results. The age of the patients in the CE and non-CE groups was 32.11 ± 4.46 and 32.33 ± 3.66 years, respectively, and there was no difference in age between the two groups (P = 0.19).

Among the 71 enrolled participants, 34 women had no history of pregnancy and delivery, including 16 CE cases and 18 non-CE cases, while there were 37 patients with pregnancy histories, including 13 CE cases and 24 non-CE cases. The Chi-square test (Chi-square value = 1.043, P = 0.307) indicated that the history of pregnancy was not associated with CE. In addition, among all the participants, 21 IVF-ET failure cases, 4 recurrent abortion patients, 4 uterine malformation patients, and 38 cases with the previous history of gynecological surgery were included, and they all presented no significant difference between the CE and non-CE groups.

### A significantly higher intrauterine microbial α-diversity was presented in CE patients

**Fig. 1:**
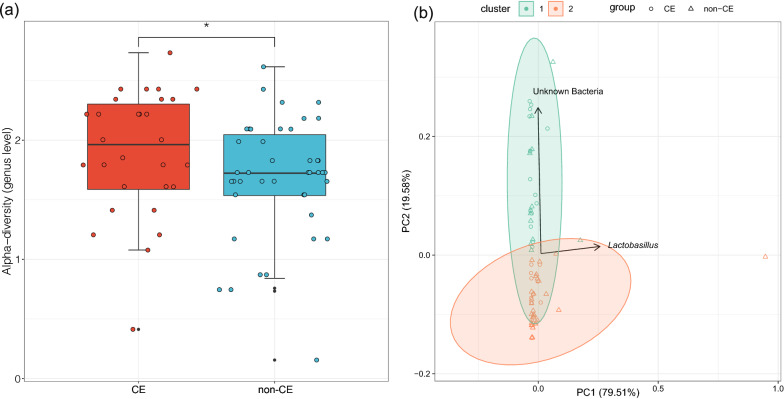
**a** The α-diversity of the uterine microbiota in the CE and non-CE groups was shown by the Shannon–Wiener index at the genus level. The red bar stands for the CE group and the blue bar stands for the non-CE group. * denotes P < 0.05. **b** The non-supervised partitioning around medoids (PAM) clustering results of the samples from CE (circle) and non-CE groups (triangle)

The α-diversity of the uterine microbiota was calculated by Shannon–Wiener index at the genus level. The results showed that the microbial α-diversity in uterine cavity in the CE group was significantly higher than that in the non-CE group (P < 0.05) (Fig. 1a). Non-supervised partitioning around medoids (PAM) algorithm was invited to explain the β-diversity of the intrauterine microbiota of two groups (Fig. 1b). The result showed that most of the samples from CE and Non-CE groups were overlapped and not separated clearly. The first principal component (PC1), mainly contributed by Lactobacillus, provided some non-CE samples with more positive values. This indicated that Lactobacillus might be a potential biomarker for distinguishing CE from non-CE samples. For the second principal component (PC2), mainly driven by unknown bacteria, CE and non-CE samples were mixed. Although CE and non-CE samples were resided significantly different microbial communities, the key taxa were still unclear. Techniques with higher resolution, such as metagenomic sequencing or whole genome sequencing, and even the cultural methods could be more appropriate options to investigate the specific genus/species in further studies. The Wilcoxon test result showed P > 0.05, suggesting that there was no significant difference between the community composition of intrauterine microbiota in CE and non-CE groups.

### The intrauterine microbial composition and abundance were different in CE patients

**Fig. 2:**
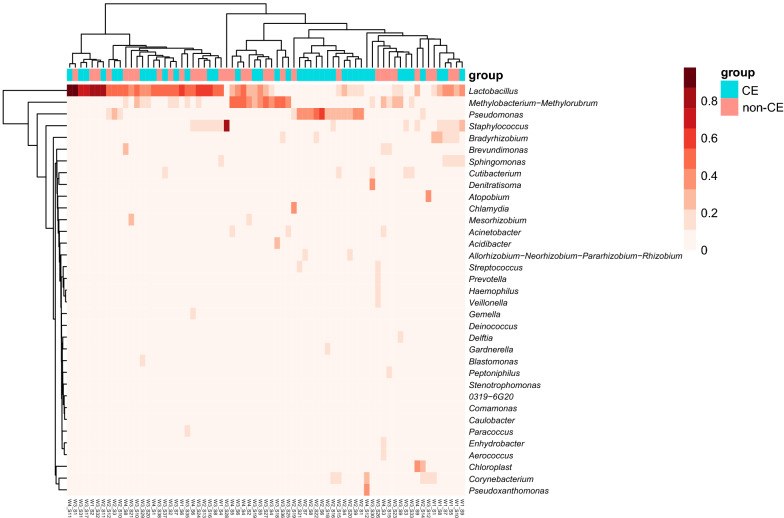
The heatmap of the microbiota in all samples at the genus level. The color from white to peony means the increasing abundance of the genus. The color bar at the top of the heatmap denotes the groups that the sample is from. Blue stands for the CE group, and red stands for the non-CE group

In general, according to the annotation and abundance at the genus level, *Lactobacillus* was the most abundant genus in all samples. It was followed by *Pseudomonas*, *Methylobacterium*, *Staphylococcus*, and *Proteobacteria* in order of abundance. However, the samples from the CE and non-CE groups could not be well-clustered into two groups (Fig. 2) indicating a similar composition of intrauterine microbiota in CE and non-CE participants.

**Fig. 3:**
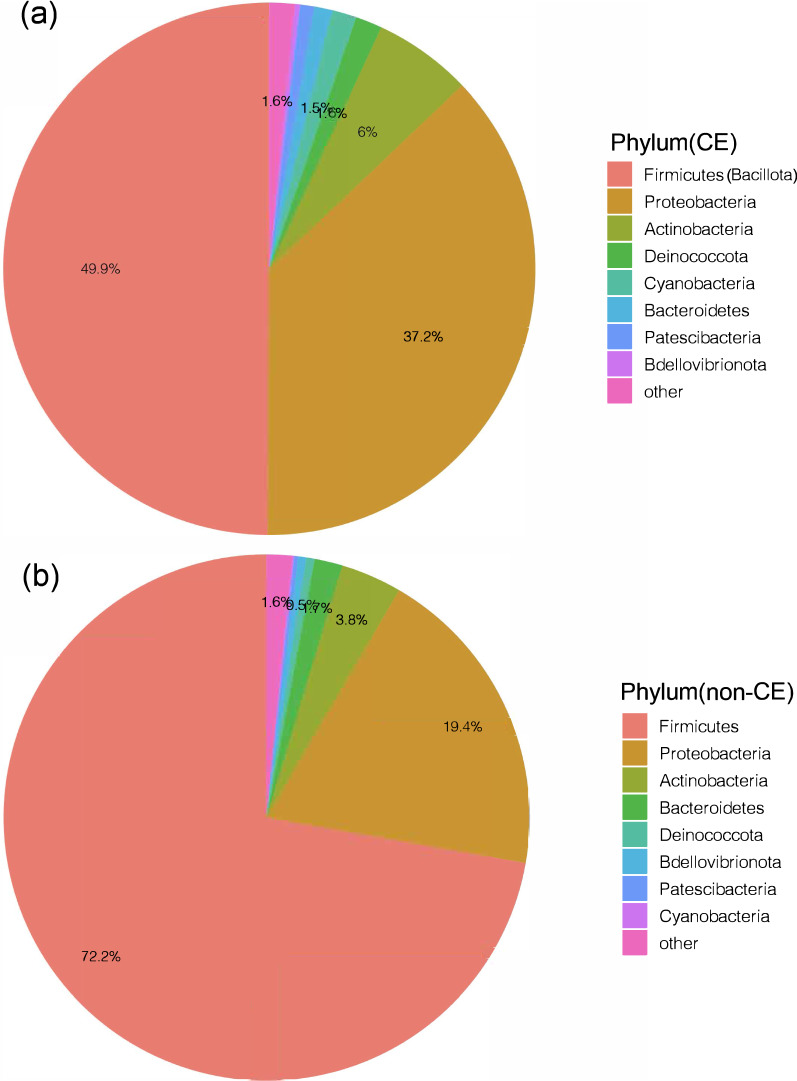
The percentage of the bacterial phylum in the uterine microbiota of the CE (**a**) and non-CE groups (**b**)

At the phylum level, Firmicutes (Bacillota) dominated in the non-CE group (identified as relative abundance ≥ 50%), accounting for 72.23% and followed by Proteobacteria (19.36%), Actinobacteriota (3.75%), Bacteroidota (1.71%), Deinococcota (0.50%), Bdellovibrionota (0.49%), Patescibacteria (0.25%), Cyanobacteria (0.10%) and others (1.59%) (Fig. 3a). In the CE group, Firmicutes (Bacillota) was the phylum with the highest abundance but could not be identified as dominant since its relative abundance (49.92%) was below 50%. The second abundant phylum was Proteobacteria, accounting for 37.19%, which was significantly higher than that in the non-CE group. The following abundant phyla were Actinobacteriota (5.98%), Deinococcota (1.62%), Cyanobacteria (1.45%), Bacteroidota (1.10%), Patescibacteria (0.86%), Bdellovibrionota (0.30%) and others (1.56%). Among them, the abundances of Actinobacteriota and Cyanobacteria in the CE group were significantly higher than those in the non-CE group (P < 0.05) (Fig. 3b). The results suggested that the abundance of Firmicutes (Bacillota) in the CE group was lower than that in the non-CE group, while the abundance of non-Firmicutes (non-Bacillota) phylum were all higher than those in non-CE samples, demonstrating that the decrease of Firmicutes (Bacillota) may be associated with CE.

**Fig. 4:**
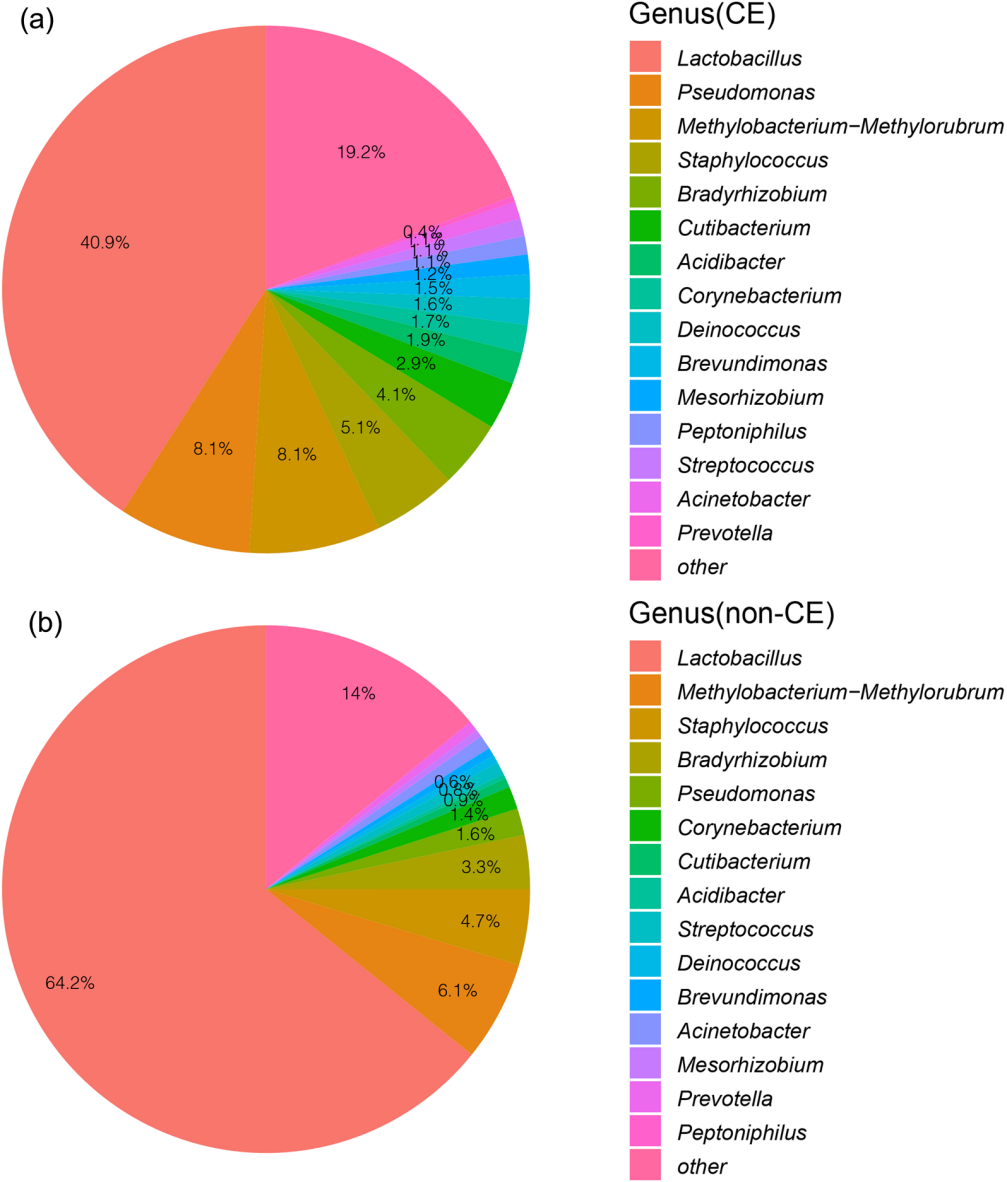
The percentage of the bacterial genus in the uterine microbiota of the CE (**a**) and non-CE groups (**b**)

The microbial community composition could be grouped into three categories at the genus level, including *Lactobacillus*, non-*Lactobacillus* (Top 15 abundant genera except for *Lactobacillus*) and others. In the non-CE group, *Lactobacillus* was identified as the dominant genus (relative abundance ≥ 50%), accounting for 64.22%. Non-*Lactobacillus* genera were *Methylobacterium-Methylorubrum* (6.13%), *Staphylococcus* (4.65%), *Bradyrhizobium* (3.30%), *Pseudomonas* (1.65%), *Corynebacterium* (1.38%), *Acinetobacter* (0.87%), *Streptococcus* (0.78%), *Prevotella* (0.58%), *Cutibacterium* (0.57%), *Deinococcus* (0.50%), *Brevundimonas* (0.50%), *Mesorhizobium* (0.46%), *Acidibacter* (0.29%), *Peptoniphilus* (0.09%) and others (14.02%) (Fig. 4a). In CE group, there was no dominant genus, and *Lactobacillus* had the highest abundance, up to 40.88%. Non-*Lactobacillus* genera were *Pseudomonas* (8.10%), *Methylobacterium-Methylorubrum* (8.06%), *Staphylococcus* (5.12%), *Bradyrhizobium* (4.12%), *Cutibacterium* (2.92%), *Acidibacter* (1.93%), *Corynebacterium* (1.71%), *Deinococcus* (1.60%), *Brevundimonas* (1.47%), *Mesorhizobium* (1.22%), *Peptoniphilus* (1.11%), *Streptococcus* (1.09%), *Acinetobacter* (1.08%), *Prevotella* (0.36%) and others (19.22%) (Fig. 4b). Compared with the non-CE group, the abundance of *Lactobacillus* significantly decreased in the CE group (P < 0.05), while the abundances of non-*Lactobacillus* and other bacteria increased in the CE group but with no statistically significant (P = 0.089 and 0.107, respectively). This suggested an important role of *Lactobacillus* played in keeping a healthy intrauterine microbial environment that is similar to the vaginal environment. What’s more, the abundances of *Pseudomonas* and *Cutibacterium* were significantly higher in the CE group (P < 0.01), which may disturb the balance of the intrauterine microbiota.

### Microbial metabolic pathways were significantly different in the intrauterine microbiota of CE patients

**Fig. 5:**
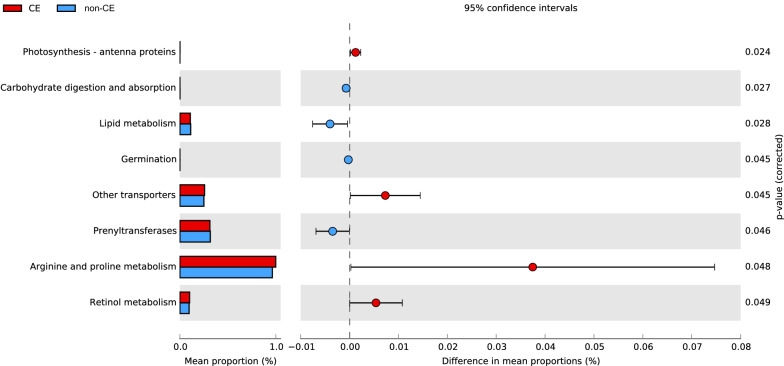
The predicted microbial metabolism pathways of the uterine microbiota of the CE and non-CE groups. The left bar stands for the mean proportion of the pathways, and the right figure shows the difference in the pathway enriched in the two groups. Blue stands for the non-CE group, and red stands for the CE group

The metabolic pathways of the intrauterine microbiota were predicted based on the microbial composition. Compared with the non-CE group, the pathways involved arginine and proline metabolism (P = 0.048) and retinol metabolism (P = 0.049) were significantly enriched in the CE group, while the metabolism of lipid (P = 0.028) and prenyltransferases (P = 0.046) were significantly decreased in the CE group (Fig. 5).

## Discussion

Currently, the diagnostic methods for CE include immunohistochemical, hysteroscopy, and detection of pathogenic microorganisms. CD138, a plasma cell surface-specific antigen, could be labeled by immunohistochemical to determine the existence of plasma cells in the endometrial matrix, which is the gold standard for CE diagnosis. However, it is difficult to distinguish using H&E staining, and relies a lot on the experiences of Laboratory physicians. Although CD138 immunohistochemical staining can improve the detection sensitivity of plasma cells, its workload is noticeable. Currently, there is no unified standard for the minimum number of plasma cells for the diagnosis of CE, which may lead to overdiagnosis [15–17]. Although CE can be diagnosed by observing the full vision of the intrauterine and finding tiny lesions through hysteroscopy, it can be affected by the hysteroscopic media, the clarity of the hysteroscopic field of vision, and the subjective judgment of laboratory physicians [18]. In addition, non-specific CE patients cannot be diagnosed independently by hysteroscopy since most of them do not present typical hysteroscopic manifestations. Additionally, the detection of pathogenic microorganisms with traditional microbial culture methods also has some limitations, such as being time consuming, a low detection rate, and a high risk of contamination [2, 19]. Detecting the pathogenic microorganisms using molecular methods, such as quantitative PCR, 16S rRNA sequencing and metagenomic sequencing, are faster and more accurate which is better than traditional cultivation methods, and they have been used in clinical diagnosis of infectious diseases, but not in CE yet. One of the reasons is lack of studies on elaborating the correlation between CE and intrauterine microbiota which can contribute to the discovery of specific microbial diagnostic markers. Moreover, in view of the clinical limitations of the above diagnostic methods for CE, in addition to the fact that CE is infected with specific pathogens, new methods for CE diagnosis based on explaining the microbiome should be developed. However, the composition of intrauterine microbiota has been reported differently so far due to the contamination from vaginal discharge that may be involved during the uterine sample collection. Hence, this study is trying to eliminate these deficiencies by comparing the distribution characteristics of the intrauterine microbiota in the CE and non-CE groups by using 16S rRNA sequencing technology, and further providing a possibility for screening the marker bacteria for diagnosis of CE, which will also guide clinical treatment of CE and the prognosis of fertility.

There was no significant difference in age distribution between the CE and non-CE groups in this study. Also, the results showed that there was no correlation between pregnancy history and CE. Previous studies have shown that obesity, oral contraceptives, history of spontaneous abortion, premature delivery and cesarean section are not risk factors for CE. Whether induced abortion history could be regarded as one of the independent risk factors for CE has still remained controversial issue [20].

Fang et al. found that the intrauterine bacteria population in patients with chronic endometritis was more diverse than that in endometrial polyps patients, and a higher proportion of *Prevotella* was also observed in patients with chronic endometritis [21]. Consistently, results in this study showed that the abundance of *Prevotella* in the CE group was higher than that in the non-CE group. Liu et al. evaluated the intrauterine microbiota of CE and non-CE patients on the seventh day after the luteinizing hormone (LH) surge by 16S rRNA sequencing. Compared with the non-CE group, the abundance of *Lactobacillus* in the CE group was significantly higher, while the abundance of non-*Lactobacillus* genera, including *Haemophilus*, *Bifidobacteria* and *Prevotella*, was lower [22]. In this study, *Lactobacillus* was the dominant bacterium in the intrauterine microbiota in the non-CE group, accounting for 64.22%. Hence, the pathogenesis of CE may be related to the decrease in the proportion of *Lactobacillus* and the increase of the proportion of non-*Lactobacillus*, suggesting that maintaining a certain abundance of *Lactobacillus* in the uterine cavity is conducive to maintaining a healthy uterine microenvironment. What’s more, among the non-*Lactobacillus* genera, it was found that the abundances of *Pseudomonas* and *Cutibacterium* were significantly higher in the CE group at the genus level (P < 0.01). Although *Pseudomonas* has been confirmed as one of the dominant bacteria in the endometrial cavity [21] and not related to a significant inflammatory immune response [23], its significantly increased abundance was found in the inflammation of the upper reproductive tract, such as endometriosis [24] and uterine pyogenesis [14], which is consistent with our results. This indicated that the overgrowth of *Pseudomonas* could disturb the balance and induce inflammatory reactions. But the related mechanisms still need further studies. *Cutibacterium* has long been considered as a common skin commensal, which contributes to acne pathogenesis and is associated with the host immune system through shaping the skin microbiota. Whereas, it has been detected in the microbiota of endometrium, vaginal fluid and amniotic fluid in recent years [25, 26], and *Cutibacterium acnes* was considered as a discriminant species of the endometrium. In addition, it has been reported that the abundance of *Cutibacterium acnes* in the endometrium is decreased in healthy pregnant women [26], while in our study, the abundance of *Cutibacterium* was significantly increased in CE patients. Since CE is one of the major driving factors of infertility, the increased abundance of *Cutibacterium* may be a potentially associated factor leading to infertility.

In our study, arginine and proline metabolism of intrauterine microbiota were significantly increased in the CE group. The disorder of the arginine metabolism can be attributed to the pathogenic factors of microorganisms [27], which may prompt the development of CE. Also, since arginine and proline metabolism in the serum and uterine tissue has been reported to relate with endometritis in mice [21], the increased microbial arginine and proline metabolism might break the homeostasis in the uterine cavity environment and further linked with the pathogenesis of CE. In addition, retinol metabolism was also enriched in the microbiota of CE patients. Retinol metabolism has been recognized as an irreplaceable pathway during endometrium regeneration by regulating the differentiation and apoptosis or senescence of endometrial cells [28, 29]. However, whether microbial retinol metabolism contributed to CE through endometrial cells still needs further studies.

The innovation of this study is the use of a self-developed uterine sampler, which has been patented and clinically verified. The use of this uterine sampler can avoid the contamination of the secretion in the vagina and cervical canal during uterine sampling. Using this method, we have reported that distinct microbial communities were colonized in cervical canal, uterus, fallopian tubes and peritoneal fluid, differing from that of the vagina, which reflects a microbiota continuum along the female reproductive tract [6]. However, the sample size was small, and the distribution and structure of CE uterine microbiota and the bacterial markers need to be further verified by expanding the research cohort. Additionally, metagenomic sequencing technology should be applied to verify the function of the microbiota.

## Conclusion

In conclusion, a certain microbial community was colonized in the uterine cavity, with *Lactobacillus* as the dominant genus, while non-*Lactobacillus* genera, such as *Methylobacterium-Methylorubrum*, *Staphylococcus*, *Bradyrhizobium*, *Pseudomonas* and *Corynebacterium* existed in low abundance. The structure and distribution of intrauterine microbiota in the CE group were different from those in the non-CE group by showing a lower abundance of *Lactobacillus* and a higher abundance of non-*Lactobacillus*, of which the abundances of *Pseudomonas* and *Cutibacterium* were significantly higher in the CE group. What’s more, microbial metabolisms of arginine, proline and retinol may be involved in the pathogenesis of CE. Whereas, research based on a larger sample size should be performed and verified in further study.

## Data Availability

The datasets generated and/or analyzed during the current study are available in the NCBI, https://www.ncbi.nlm.nih.gov/bioproject/839449.

## Abbreviations

CE: Chronic endometritis
IVF-ET: In vitro fertilization and embryo transfer
HPF: High power field
OTUs: Operational taxonomic units
PAM: Partitioning around medoids

## References

[1] Song D, Li T-C, Zhang Y, Feng X, Xia E, Huang X, Xiao Y (2019). Correlation between hysteroscopy findings and chronic endometritis. Fertil Steril.

[2] Moreno I, Cicinelli E, Garcia-Grau I, Gonzalez-Monfort M, Bau D, Vilella F, De Ziegler D, Resta L, Valbuena D, Simon C (2018). The diagnosis of chronic endometritis in infertile asymptomatic women: a comparative study of histology, microbial cultures, hysteroscopy, and molecular microbiology. Am J Obstet Gynecol.

[3] Kitaya K, Takeuchi T, Mizuta S, Matsubayashi H, Ishikawa T (2018). Endometritis: new time, new concepts. Fertil Steril.

[4] Cicinelli E, Matteo M, Trojano G, Mitola PC, Tinelli R, Vitagliano A, Crupano FM, Lepera A, Miragliotta G, Resta L (2018). Chronic endometritis in patients with unexplained infertility: prevalence and effects of antibiotic treatment on spontaneous conception. Am J Reprod Immunol.

[5] Kitaya K, Matsubayashi H, Takaya Y, Nishiyama R, Yamaguchi K, Takeuchi T, Ishikawa T (2017). Live birth rate following oral antibiotic treatment for chronic endometritis in infertile women with repeated implantation failure. Am J Reprod Immunol.

[6] Chen C, Song X, Wei W, Zhong H, Dai J, Lan Z, Li F, Yu X, Feng Q, Wang Z (2017). The microbiota continuum along the female reproductive tract and its relation to uterine-related diseases. Nat Commun.

[7] Cicinelli E, De Ziegler D, Nicoletti R, Colafiglio G, Saliani N, Resta L, Rizzi D, De Vito D (2008). Chronic endometritis: correlation among hysteroscopic, histologic, and bacteriologic findings in a prospective trial with 2190 consecutive office hysteroscopies. Fertil Steril.

[8] Park HJ, Kim YS, Yoon TK, Lee WS (2016). Chronic endometritis and infertility. Clin Exp Reprod Med.

[9] Bouet P-E, El Hachem H, Monceau E, Gariépy G, Kadoch I-J, Sylvestre C (2016). Chronic endometritis in women with recurrent pregnancy loss and recurrent implantation failure: prevalence and role of office hysteroscopy and immunohistochemistry in diagnosis. Fertil Steril.

[10] Ye Y (2011). Identification and quantification of abundant species from pyrosequences of 16S rRNA by consensus alignment. IEEE Int Conf Bioinformatics Biomed.

[11] Caporaso JG, Kuczynski J, Stombaugh J, Bittinger K, Bushman FD, Costello EK, Fierer N, Peña AG, Goodrich JK, Gordon JI (2010). QIIME allows analysis of high-throughput community sequencing data. Nat Methods.

[12] Bolyen E, Rideout JR, Dillon MR, Bokulich NA, Abnet CC, Al-Ghalith GA, Alexander H, Alm EJ, Arumugam M, Asnicar F (2019). Reproducible, interactive, scalable and extensible microbiome data science using QIIME 2. Nat Biotechnol.

[13] Quast C, Pruesse E, Yilmaz P, Gerken J, Schweer T, Yarza P, Peplies J, Glöckner FO (2013). The SILVA ribosomal RNA gene database project: improved data processing and web-based tools. Nucleic Acids Res.

[14] Parks DH, Tyson GW, Hugenholtz P, Beiko RG (2014). STAMP: statistical analysis of taxonomic and functional profiles. Bioinformatics.

[15] Kasius J, Broekmans F, Sie-Go D, Bourgain C, Eijkemans M, Fauser B, Devroey P, Fatemi H (2012). The reliability of the histological diagnosis of endometritis in asymptomatic IVF cases: a multicenter observer study. Hum Reprod.

[16] McQueen DB, Perfetto CO, Hazard FK, Lathi RB (2015). Pregnancy outcomes in women with chronic endometritis and recurrent pregnancy loss. Fertil Steril.

[17] Cicinelli E, De Ziegler D, Nicoletti R, Tinelli R, Saliani N, Resta L, Bellavia M, De Vito D (2009). Poor reliability of vaginal and endocervical cultures for evaluating microbiology of endometrial cavity in women with chronic endometritis. Gynecol Obstet Invest.

[18] Cicinelli E, Vitagliano A, Kumar A, Lasmar RB, Bettocchi S, Haimovich S, Kitaya K, de Ziegler D, Simon C, Moreno I (2019). Unified diagnostic criteria for chronic endometritis at fluid hysteroscopy: proposal and reliability evaluation through an international randomized-controlled observer study. Fert Ster.

[19] Vitagliano A, Saccardi C, Noventa M, Sardo ADS, Saccone G, Cicinelli E, Pizzi S, Andrisani A, Litta PS (2018). Effects of chronic endometritis therapy on in vitro fertilization outcome in women with repeated implantation failure: a systematic review and meta-analysis. Fert Ster.

[20] Chen Y-q, Fang R-l, Luo Y-n, Luo C-q (2016). Analysis of the diagnostic value of CD138 for chronic endometritis, the risk factors for the pathogenesis of chronic endometritis and the effect of chronic endometritis on pregnancy: a cohort study. BMC Womens Health.

[21] Fang R-L, Chen L-X, Shu W-S, Yao S-Z, Wang S-W, Chen Y-Q (2016). Barcoded sequencing reveals diverse intrauterine microbiomes in patients suffering with endometrial polyps. Am J Transl Res.

[22] Liu Y, Ko EY-L, Wong KK-W, Chen X, Cheung W-C, Law TS-M, Chung JP-W, Tsui SK-W, Li T-C, Chim SS-C (2019). Endometrial microbiota in infertile women with and without chronic endometritis as diagnosed using a quantitative and reference range-based method. Fert Ster.

[23] Mitchell CM, Haick A, Nkwopara E, Garcia R, Rendi M, Agnew K, Fredricks DN, Eschenbach D (2015). Colonization of the upper genital tract by vaginal bacterial species in nonpregnant women. Am J Obstet Gynecol.

[24] Wei W, Zhang X, Tang H, Zeng L, Wu R (2020). Microbiota composition and distribution along the female reproductive tract of women with endometriosis. Ann Clin Microbiol Antimicrob.

[25] He Q, Kwok L-Y, Xi X, Zhong Z, Ma T, Xu H, Meng H, Zhao F, Zhang H (2020). The meconium microbiota shares more features with the amniotic fluid microbiota than the maternal fecal and vaginal microbiota. Gut Microbes.

[26] Riganelli L, Iebba V, Piccioni M, Illuminati I, Bonfiglio G, Neroni B, Calvo L, Gagliardi A, Levrero M, Merlino L (2020). Structural variations of vaginal and endometrial microbiota: hints on female infertility. Front Cell Infect Microbiol.

[27] E3S web of conferences: 2021 EDP sciences; 2021: 03017.

[28] Kuroda K (2019). Impaired endometrial function and unexplained recurrent pregnancy loss. Hypertens Res Pregnancy.

[29] Mao Y, Wang M, Xiong Y, Wen X, Zhang M, Ma L, Zhang Y. MELTF might regulate ferroptosis, pyroptosis, and autophagy in platelet-rich plasma-mediated endometrial epithelium regeneration. 2022. 10.1007/s43032-022-01101-y 36303086

